# Diet and Living Environment as Novel Etiological Factors for Melasma: The Results Form a Retrospective Case–Control Study of 150 Chinese Patients

**DOI:** 10.1111/jocd.70038

**Published:** 2025-02-11

**Authors:** Yue Shi, Shun Guo, Cheng Tan

**Affiliations:** ^1^ Department of Dermatology Affiliated Hospital of Nanjing University of Chinese Medicine Nanjing China

**Keywords:** case–control study, diet, etiological factors, living environment, melasma

## Abstract

**Background:**

Melasma, a condition with complex and multifactorial pathogenesis, has traditionally been linked to factors such as ultraviolet (UV) exposure, hormonal changes, and genetic predisposition. Despite societal advances and shifts in lifestyle, updates on causal factors remain limited. Our study aims to address this gap by investigating emerging pathogenic factors that differ from those conventionally reported.

**Objective:**

We conducted a case–control study to explore novel etiological factors contributing to the onset of melasma in Chinese patients, with an emphasis on how these differ from previously established causes.

**Methods:**

The study included 150 patients (144 women and 6 men) diagnosed with melasma at Jiangsu Province Hospital of Chinese Medicine from April to October 2023. A control cohort of individuals without melasma was enrolled from the community. Demographic and clinical characteristics were collected, along with exogenous factors and histories of related dermatoses and non‐dermatoses, through a questionnaire administered with the guidance of a dermatologist. Etiological factors were assessed using univariate analysis followed by multivariate analysis.

**Results:**

Overall, our results were consistent with previous studies. In addition, alcohol intake (OR: 20.05, 95% CI: 1.17–343.17) and breast cystic hyperplasia (OR: 15.98, 95% CI: 4.28–59.72) were found to be novel triggering factors of melasma, whereas change of residence (OR: 0.03, 95% CI: 0.00–0.30), house renovation (OR: 0.13, 95% CI: 0.03–0.58) and soft drink intake (OR: 0.04, 95% CI: 0.00–0.83) were protective.

**Conclusion:**

These findings suggest that diet and the living environment are novel etiological factors for melasma.

## 1 Introduction

Melasma is a chronic acquired hypermelanosis, characterized by symmetric brown patches on sun‐exposed areas, particularly the face [1]. Its global prevalence ranges from 1.5% to 50%, with higher rates observed in individuals with darker skin types and in regions with high UV exposure [2]. The condition predominantly affects women and can significantly impact their quality of life [3]. While the exact pathogenesis of melasma remains unclear, several epidemiological factors—such as pregnancy, sun exposure, hormonal changes, genetic predisposition, inflammatory skin conditions, and the use of photosensitive medications—have been identified as modifiable risk factors in previous studies [1, 4, 5, 6].

Histopathologically, melasma is characterized by increased pigmentation in the epidermis and/or dermis, enlarged melanocytes, and evidence of solar elastosis [2]. These findings suggest that melasma may be preventable through lifestyle modifications. However, with societal development and changes in people's daily habits, additional factors—such as diet and environmental conditions—might also contribute to the incidence of melasma. Despite the potential importance of these factors, few epidemiological case–control studies have systematically explored their relationship to the onset of this skin condition.

In the present study, we retrospectively analyzed the demographic and clinical characteristics, as well as the risk and protective factors associated with melasma in a population with and without the condition. By doing so, we aim to identify novel contributing factors that may influence the incidence of melasma and better understand its complex etiology.

## 2 Material and Methods

This study was a case–control study involving patients 40 diagnosed with melasma between April 2023 and October 2023 in the dermatology outpatient clinic of Jiangsu Province Hospital of Chinese Medicine. The inclusion criteria were as follows: (1) patients with melasma confirmed by two fully qualified dermatologists during medical consultation; (2) related exposure factors were available; and (3) patients willing to give informed consent. The exclusion criteria included: (1) patients with psychological disorders; (2) patients diagnosed with severe cardiovascular, cerebrovascular, and hematopoietic diseases; and (3) patients with inadequate hepatic or renal function. Subsequently, by matching patient gender and age, we selected participants without melasma from the community in our institution as the control cohort. The Jiangsu Province Hospital of Chinese Medicine Ethics Committee approved the study protocol (Ethical approval No. 2014NL‐024‐02), and all study subjects signed the informed consent form. Both demographic and clinical characteristics of eligible patients were collected. Related pathogenic factors, including exogenous factors, relative dermatosis and non‐dermatosis history were also obtained through a questionnaire survey with the guidance of a dermatologist without blinding. Similarly, these factors were investigated in control cohort cases using the same method and questionnaire. Data were entered into MS Excel 2016 tables and analyzed using IBM SPSS 25.0 software. Continuous variable and dichotomous variable were compared using unpaired *t* test and chi‐square test, respectively. Pathogenic factors of melasma were summarized using univariate analysis followed by multivariate analysis. Significance was considered at *p* ≤ 0.05.

## 3 Results

### 3.1 Demographic and Clinical Characteristics of Enrolled Patients

In total, 150 patients with melasma, including 144 (96.00%) female cases, were enrolled in the present study as case cohort. The response rate of this group was 93.75% (150/160). Its demographic and clinical characteristics are shown in Table 1.

**TABLE 1:** Demographic and clinical characteristics of enrolled patients (*n* = 150).

Variable	Values
Gender‐*N* (%)	
Male	6 (4.00%)
Female	144 (96.00%)
Age‐mean (SD) in years	39.98 (6.01)
Course‐median (p25‐p75) in years	4.0 (2.0–7.0)
Fitzpatrick phototypes—*N* (%)	
II	50 (33.33%)
III	72 (48.00%)
IV	28 (18.67%)
Family with melasma—*N* (%)	44 (29.33%)
First degree relative	38 (25.33%)
Second degree relatives	6 (4.00%)
Lesion affected—*N* (%)	
Zygomatic	105 (70.00%)
Cheek	101 (67.33%)
Temporal	61 (40.67%)
Nasal	50 (33.33%)
Frontal	49 (32.67%)
Orbital	40 (26.67%)
Perilabial	28 (18.67%)
Mandibular	13 (8.67%)
Parotid	17 (11.33%)
Glabellar	8 (5.33%)
Extrfacial	—
Morphology of lesions—*N* (%)	
Patchy	76 (50.67%)
Mixed	58 (38.67%)
Spotted	16 (10.67%)
Band	—
Clinical melasma classification—*N* (%)	
Centrofacial	82 (54.67%)
Peripheral	47 (31.33%)
Mixed	21 (14.00%)
MASI‐median (p25–p75)	7.5 (4.5–12.0)

Abbreviation: MASI, Melasma Area Severity Index.

The patients had a mean age of 39.98 years, a median course of 4.00 years, and a positive family history of melasma of 29.33% (*n* = 44), especially for first degree relative (*n* = 38, 25.33%). The most common fitzpatrick phototype was type III (*n* = 72, 48.00%) existed among these patients.

The analysis of the characteristics of their facial lesion revealed that zygomatic (70%) and cheek (67.33%) were the most commonly affected parts. In addition, 50.67% of the morphology of lesions was patchy with a centrofacial topographical preference of 54.67% (*n* = 82). Their median melasma area severity index (MASI) was 7.5. In total, 142 participants without melasma were enrolled as case controls. The response rate was 88.75% (142/160), which was no statistically significantly different from that of the case cohort (*p* > 0.05). There was no significant difference between the two cohorts in gender, age and body mass index (BMI) (Table 2).

**TABLE 2:** Univariate analysis of exogenous factors for melasma.

Factors	Case (*n* = 150)	Control (*n* = 142)	OR (95% CI)	*p*
	*N* (%)	*N* (%)		
Gender				
Male	6 (4.0%)	12 (8.5%)	0.45 (0.17–1.24)	0.122
Female	144 (96.0%)	130 (91.5%)		
Age‐mean ± SD, in years	39.98 ± 6.01	38.44 ± 7.96	1.03 (1.00–1.07)	0.064
BMI, kg/m^2^	21.51 ± 2.18	21.74 ± 2.54	0.96 (0.87–1.06)	0.406
Menstrual cycle				
Normal	44 (30.6%)	80 (61.5%)	3.46 (2.14–5.60)	0.000
Abnormal	100 (69.4%)	50 (38.5%)		
HRT				
With	18 (12.5%)	9 (7.4%)	2.05 (0.89–4.74)	0.093
Without	122 (84.7%)	113 (92.6%)		
Combined oral contraceptives using				
Yes	43 (30.7%)	27 (20.8%)	1.73 (1.00–2.98)	0.049
No	97 (69.3%)	103 (79.2%)		
Previous sunburn or exposure to sun ≥ 2 h/d				
Yes	98 (65.3%)	29 (20.4%)	7.34 (4.33–12.46)	0.000
No	52 (34.7%)	113 (79.6%)		
Exposure to flood light, strong heat source or artificial lighting equipment ≥ 2 h/day				
Yes	76 (50.7%)	88 (62.0%)	0.63 (0.40–1.00)	0.052
No	74 (49.3%)	54 (38.0%)		
Regular sunscreen cream using				
Yes	99 (66.0%)	63 (44.4%)	2.43 (1.52–3.91)	0.000
No	51 (34.0%)	79 (55.6%)		
History of face laser treatment				
Yes	37 (24.7%)	36 (25.4%)	0.96 (0.57–1.64)	0.892
No	113 (75.3%)	106 (74.6%)		
Habit of rubbing the face				
Yes	47 (31.1%)	30 (21.1%)	1.70 (1.00–2.90)	0.049
No	103 (68.7%)	112 (78.9%)		
Mental pressure				
Yes	94 (62.7%)	65 (45.8%)	1.99 (1.25–3.17)	0.004
No	56 (37.3%)	77 (54.2%)		
Insomnia or staying up late				
Yes	91 (60.7%)	64 (45.1%)	1.88 (1.18–2.99)	0.008
No	59 (39.3%)	78 (54.9%)		
Smoking or passive smoking				
Yes	47 (31.3%)	42 (29.6%)	1.09 (0.66–1.79)	0.745
No	103 (68.7%)	100 (70.4%)		
Alcohol intake				
Yes	18 (12.0%)	5 (3.5%)	3.74 (1.35–10.35)	0.011
No	132 (88.0%)	137 (96.5%)		
Coffee intake				
Yes	16 (10.7%)	27 (19.0%)	0.50 (0.26–0.99)	0.047
No	134 (89.3%)	115 (81.0%)		
Soft drink intake				
Yes	1 (0.7%)	29 (20.4%)	0.03 (0.00–0.20)	0.000
No	149 (99.3%)	113 (79.6%)		
Regular physical exercise				
Yes	40 (26.7%)	60 (42.3%)	0.50 (0.30–0.81)	0.005
No	110 (73.3%)	82 (57.7%)		
House renovation				
Yes	49 (32.7%)	74 (52.1%)	0.45 (0.28–0.72)	0.001
No	101 (67.3%)	68 (47.9%)		
Driving				
Yes	60 (40.0%)	50 (35.2%)	1.23 (0.76–1.97)	0.399
No	90 (60.0%)	92 (64.8%)		
Habit of steaming hot sauna				
Yes	12 (8.0%)	7 (4.9%)	1.68 (0.64–4.39)	0.292
No	138 (92.0%)	135 (95.3%)		
Dry facial skin				
Yes	105 (70.0%)	94 (66.2%)	1.19 (0.73–1.95)	0.486
No	45 (30.0%)	48 (33.8%)		
Gain weight				
Yes	26 (17.3%)	31 (21.8%)	0.75 (0.42–1.34)	0.333
No	124 (82.7%)	111 (78.2%)		
Lose weight				
Yes	6 (4.0%)	9 (6.3%)	0.62 (0.21–1.78)	0.370
No	144 (96.0%)	133 (93.7%)		
History of facial trauma				
Yes	5 (3.3%)	21 (14.8%)	0.20 (0.07–0.54)	0.002
No	145 (96.7%)	121 (85.2%)		
Change of residence				
Yes	6 (4.0%)	50 (35.2%)	0.08 (0.03–0.19)	0.000
No	144 (96.0%)	92 (64.8%)		
Family history				
Yes	44 (29.5%)	33 (23.2%)	1.38 (0.82–2.34)	0.225
No	105 (70.5%)	109 (76.8%)		

Abbreviations: BMI, Body Mass Index; HRT, hormone replacement therapy.

**TABLE 3:** Univariate analysis of relative dermatosis for melasma.

Diseases	Case (*n* = 150)	Control (*n* = 142)	OR (95% CI)	*p*
	*N* (%)	*N* (%)		
Hori's nevus				
With	24 (16.0%)	6 (4.2%)	4.32 (1.71–10.91)	0.002
Without	126 (84.0%)	136 (95.8%)		
Pigmentation of conjunctiva				
With	50 (33.3%)	3 (2.1%)	23.17 (7.03–76.38)	0.000
Without	100 (66.7%)	139 (97.9%)		
Pigmented fungiform papillae of the tongue				
With	44 (29.3%)	1 (0.7%)	58.53 (7.94–431.63)	0.000
Without	106 (70.7%)	141 (99.3%)		
Lentigo				
With	18 (12.0%)	35 (24.6%)	0.42 (0.22–0.78)	0.006
Without	132 (88.0%)	107 (75.4%)		
Nevoid lentigo				
With	4 (2.7%)	8 (5.6%)	0.46 (0.14–1.56)	0.212
Without	146 (97.3%)	134 (94.4%)		
Seborrheic keratosis				
With	18 (12.0%)	9 (6.3%)	2.02 (0.87–4.65)	0.100
Without	132 (88.0%)	133 (93.7%)		
Idiopathic guttate Hypomelanosis				
With	4 (2.7%)	2 (1.4%)	1.92 (0.35–10.64)	0.456
Without	146 (97.3%)	140 (98.6%)		
Achromic nevus				
With	2 (1.3%)	2 (1.4%)	0.95 (0.13–6.81)	0.956
Without	148 (98.7%)	140 (98.6%)		
Rosacea				
With	1 (0.7%)	6 (4.2%)	0.15 (0.02–1.28)	0.083
Without	149 (99.3%)	136 (95.8%)		
Acne				
With	62 (41.3%)	51 (35.9%)	1.26 (0.78–2.02)	0.342
Without	88 (58.7%)	91 (64.1%)		
Seborrheic dermatitis				
With	12 (8.0%)	24 (16.9%)	0.43 (0.21–0.89)	0.024
Without	138 (92.0%)	118 (83.1%)		
Eczema				
With	23 (15.3%)	15 (10.6%)	1.53 (0.77–3.07)	0.228
Without	127 (84.7%)	127 (89.4%)		
Lichen simplex chronicus				
With	6 (4.0%)	15 (10.6%)	0.35 (0.13–0.94)	0.036
Without	144 (96.0%)	127 (89.4%)		
Androgenetic alopecia				
With	23 (15.3%)	14 (9.9%)	1.65 (0.82–3.36)	0.163
Without	127 (84.7%)	128 (90.1%)		
Flat warts				
With	15 (10.0%)	6 (4.2%)	2.52 (0.95–6.68)	0.064
Without	135 (90.0%)	136 (95.8%)		
Syringoma				
With	8 (5.3%)	3 (2.1%)	2.61 (0.68–10.04)	0.163
Without	142 (94.7%)	139 (97.9%)		
Cherry angiomas				
With	4 (2.7%)	5 (3.5%)	0.75 (0.19–2.85)	0.674
Without	146 (97.3%)	137 (96.5%)		

### 3.2 Univariate Analysis of Factors for Melasma

In the present study, 53 factors, including 28 exogenous factors, 17 relative dermatosis, and 10 non‐dermatosis, were compared and analyzed between the two cohorts.

Univariate analysis revealed that irregular menstrual cycle (OR: 3.46, 95% CI: 2.14–5.60), combined oral contraceptives using (OR: 1.73, 95% CI: 1.00–2.98), previous sunburn or exposure to sun ≥ 2 h/day (OR: 7.34, 95% CI: 4.33–12.46), regular sunscreen cream using (OR: 2.43, 95% CI: 1.52–3.91), habit of rubbing the face (OR: 1.70, 95% CI: 1.00–2.90), mental pressure (OR: 1.99, 95% CI: 1.25–3.17), insomnia or staying up late (OR: 1.88, 95% CI: 1.18–2.99), alcohol intake (OR: 3.74, 95% CI: 1.35–10.35) were positively correlated with melasma incidence. Moreover, we found that patients with Hori's nevus (OR: 4.32, 95% CI: 1.71–10.91), pigmentation of conjunctiva (OR: 23.17, 95% CI: 7.03–76.38), pigmented fungiform papillae of the tongue (OR: 58.53, 95% CI: 7.94–431.63), breast cystic hyperplasia (OR: 6.45, 95% CI: 3.83–10.86), hysteromyoma (OR: 1.82, 95% CI: 1.02–3.24) or adnexitis (OR: 2.90, 95% CI: 1.11–7.57) had a higher risk of melasma.

In addition, using regular cosmetics or skin care products (OR: 0.43, 95% CI: 0.23–0.78), coffee intake (OR: 0.50, 95% CI: 0.26–0.99), soft drink intake (OR: 0.03, 95% CI: 0.00–0.20), regular physical exercise (OR: 0.50, 95% CI: 0.30–0.81), house renovation (OR: 0.45, 95% CI: 0.28–0.72), history of facial trauma (OR: 0.20, 95% CI: 0.07–0.54), change of residence (OR: 0.08, 95% CI: 0.03–0.19) decreased the risk of melasma. Besides, previous diagnosis with lentigo (OR: 0.42, 95% CI: 0.22–0.78), seborrheic dermatitis (OR: 0.43, 95% CI: 0.21–0.89) or lichen simplex chronicus (OR: 0.35, 95% CI: 0.13–0.94) was negatively correlated with melasma morbidity (Tables 2, 3, 4).

**TABLE 4:** Univariate analysis of relative non‐dermatosis for melasma.

Diseases	Case (*n* = 150)	Control (*n* = 142)	OR (95% CI)	*p*
	*N* (%)	*N* (%)		
Breast cystic hyperplasia				
With	119 (79.3%)	53 (37.3%)	6.45 (3.83–10.86)	0.000
Without	31 (20.7%)	89 (62.7%)		
Hyperthyroidism				
With	2 (1.3%)	1 (0.7%)	1.91 (0.17–21.45)	0.600
Without	148 (98.7%)	141 (99.3%)		
Hypothyroidism				
With	1 (0.7%)	1 (0.7%)	0.95 (0.05–15.27)	0.969
Without	149 (99.3%)	141 (99.3%)		
Hepatic lipidosis				
With	2 (1.3%)	5 (3.5%)	0.37 (0.07–1.94)	0.240
Without	148 (98.7%)	137 (96.5%)		
Hysteromyoma				
With	39 (27.1%)	21 (16.1%)	1.82 (1.02–3.24)	0.042
Without	105 (72.9%)	109 (83.9%)		
Ovarian cyst				
With	10 (6.9%)	11 (8.5%)	0.77 (0.32–1.85)	0.565
Without	134 (93.1%)	119 (91.5%)		
Adnexitis				
With	17 (11.8%)	5 (3.8%)	2.90 (1.11–7.57)	0.030
Without	127 (88.2%)	125 (96.2%)		
Endometriosis				
With	1 (0.7%)	6 (4.6%)	1.52 (0.18–1.28)	0.083
Without	143 (99.3%)	124 (95.4%)		
PCOS				
With	1 (0.7%)	4 (3.1%)	0.23 (0.03–2.10)	0.193
Without	143 (99.3%)	126 (96.9%)		
Hypertension				
With	6 (4.0%)	5 (3.5%)	1.42 (0.34–3.83)	0.830
Without	144 (96.0%)	137 (96.5%)		

Abbreviation: PCOS, polycystic ovarian syndrome.

### 3.3 Multivariate Analysis of Factors for Melasma

Significant factors identified from univariate analysis were used in multivariate analysis to determine the factors driving melasma incidence. The results revealed that irregular menstrual cycle (OR: 4.32, 95% CI: 1.28–14.50), previous sunburn or exposure to sun ≥ 2 h/day (OR: 19.43, 95% CI: 4.72–79.99), regular sunscreen cream using (OR: 6.81, 95% CI: 1.64–28.19) and alcohol intake (OR: 20.05, 95% CI: 1.17–343.17) increased the risk of melasma. Additionally, change of residence (OR: 0.03, 95% CI: 0.00–0.30), house renovation (OR: 0.13, 95% CI: 0.03–0.58) and soft drink intake (OR: 0.04, 95% CI: 0.00–0.83) were confirmed as protective factors.

Particularly, breast cystic hyperplasia (OR: 15.98, 95% CI: 4.28–59.72) might elevate the incidence risk of melasma in patients, whereas lentigo (OR: 0.08, 95% CI: 0.01–0.52) could be a protective factor (Table 5).

**TABLE 5:** Multivariate analysis of factors for melasma.

Factors	aOR(95% CI)	*p*
Menstrual cycle	4.32 (1.28–14.50)	0.018
Previous sunburn or exposure to sun ≥ 2 h/day	19.43 (4.72–79.99)	0.000
Regular sunscreen cream using	6.81 (1.64–28.19)	0.008
Alcohol intake	20.05 (1.17–343.17)	0.039
Change of residence	0.03 (0.00–0.30)	0.002
Hori's nevus	15.53 (0.81–296.80)	0.068
Pigmentation of conjunctiva	9.23 (0.77–110.21)	0.079
Pigmented fungiform papillae of the tongue	172.78 (0.13–227559.09)	0.160
Lentigo	0.08 (0.01–0.52)	0.008
Seborrheic dermatitis	0.21 (0.03–1.39)	0.107
Lichen simplex chronicus	0.11 (0.01–1.25)	0.075
Breast cystic hyperplasia	15.98 (4.28–59.72)	0.000
Hysteromyoma	1.49 (0.34–6.56)	0.598
Adnexitis	1.70 (0.12–23.84)	0.693
Soft drink intake	0.04 (0.00–0.83)	0.037
Combined oral contraceptives using	1.13 (0.26–4.95)	0.871
Regular cosmetics or Skin care products using	0.22 (0.04–1.29)	0.093
Habit of rubbing the face	0.90 (0.24–3.43)	0.878
Pressure	3.50 (0.92–13.37)	0.066
Insomnia or staying up late	3.47 (0.97–12.46)	0.056
Coffee intake	0.47 (0.06–3.39)	0.452
Regular physical exercise	0.59 (0.16–2.08)	0.409
History of facial trauma	0.19 (0.01–2.43)	0.199
House renovation	0.13 (0.03–0.58)	0.008

Abbreviation: aOR, adjusted OR.

## 4 Discussion

This study confirmed that sun exposure and sex hormones are key risk factors for melasma, aligning with the findings of previous studies [7, 8, 9]. In particular, we identified novel factors such as alcohol intake and a history of breast cystic hyperplasia as additional contributors to melasma risk.

Notably, this is the first study to demonstrate a strong positive relationship between alcohol intake and melasma. Although this association is compelling, further research is necessary to clarify the underlying mechanisms. Matsumoto et al. [10] showed that ethanol intake can induce skin hyperpigmentation in an aldehyde dehydrogenase 2 activity‐dependent manner in vivo, with the degree of pigmentation being concentration‐dependent. Additionally, other studies [11, 12] have reported that ethanol consumption exacerbates UV exposure‐induced hyperpigmentation, particularly in patients with primary biliary cirrhosis and alcohol‐related liver disease. These studies suggest that the pigmentation disorder, which is characterized by excess melanin in giant melanosomes with a normal number of melanocytes, shares pathogenic similarities with melasma. Based on this evidence, alcohol intake may act as a trigger for melasma, potentially through liver dysfunction linked to alcohol consumption.

Similarly, breast cystic hyperplasia, a condition more common in women, is associated with factors such as stress, abnormal hormone levels, and genetic predisposition [13]. Interestingly, these factors have also been identified as triggers for melasma [9], indicating potential commonalities in their pathogenesis.

Another significant finding of our study was the association between changing residence and house renovations with a reduced risk of melasma. This may be explained by relocation from more polluted areas to cities with healthier environments, likely driven by economic improvement. Pollution, particularly particulate matter (PM) and polycyclic aromatic hydrocarbons (PAHs) is a well‐documented contributor to oxidative stress and skin disorders. Roberts et al. [14] noted that these pollutants penetrate the skin and generate reactive oxygen species (ROS), exacerbating melasma. The interplay between ROS, ultraviolet (UV) exposure, and genetic factors increases metalloproteinase activity, leading to melasma development. This suggests that environmental improvements can mitigate melasma risk by reducing pollutant‐induced oxidative stress and inflammation.

The observed protective effect of soft drink consumption may be linked to the availability of vitamin‐enriched beverages, particularly those containing vitamin C (VC). VC has well‐established antimelanogenic properties, as it inhibits tyrosinase activity and reduces oxidative stress in melanocytes. Clinical studies demonstrate that VC can significantly improve melasma symptoms, as evidenced by reductions in pigmentation following iontophoresis treatments [15]. Additionally, VC and its derivatives induce intramelanocytic acidification, further suppressing melanogenesis without altering melanogenic protein expression [16]. Dietary antioxidants, including VC, have also been shown to lower melanin levels and prevent hyperpigmentation. These findings align with previous research supporting the role of VC in managing pigmentation disorders, highlighting its potential in fortified beverages to offer protective benefits against melasma.

Conversely, the relationship between regular sunscreen use and increased melasma incidence observed in this study may appear contradictory to established knowledge. While sunscreen is widely recommended for UV protection, its efficacy in reducing pigmentation disorders such as melasma depends on proper usage. Inadequate application or reliance solely on sunscreen without additional physical barriers (e.g., hats or shade) might fail to prevent UV‐induced pigmentation effectively. Moreover, this finding could reflect reverse causation, where individuals with melasma are more likely to use sunscreen after diagnosis. The complexity of sunscreen's role in skin conditions is supported by conflicting findings in the literature. For example, while some studies suggest a potential link between sunscreen use and certain dermatological conditions, such as frontal fibrosing alopecia (FFA), others argue that biases and confounding factors complicate interpretations [17, 18]. Despite these inconsistencies, sunscreen use remains a widely endorsed strategy for skin cancer prevention and melasma management.

Environmental factors also play a pivotal role in melasma's pathogenesis. Air pollution, particularly in urban areas, is a significant contributor to skin hyperpigmentation. Recent studies highlight that PM and PAHs exacerbate UV radiation's harmful effects, accelerating skin aging and pigmentation [14, 19]. PM2.5, a specific type of fine particulate matter, activates the aryl hydrocarbon receptor (AhR), potentially upregulating melanogenesis pathways [20]. This synergistic interaction between pollution and UV exposure underscores the importance of environmental quality in mitigating melasma risk. Relocation to areas with lower pollution levels may reduce these synergistic effects, highlighting the protective role of an improved living environment.

These findings collectively underscore the multifactorial nature of melasma and the importance of systemic, environmental, and lifestyle factors in its development. Addressing these factors may lead to improved preventive and therapeutic strategies for this condition.

In recent years, treatment strategies for melasma have primarily centered around topical agents, chemical peels, and laser therapies. However, these methods often come with significant limitations, including variable efficacy, potential side effects, and a high recurrence rate. These challenges underscore the urgent need to identify new risk factors that could help develop more effective prevention and treatment strategies. Our study highlights the potential role of diet and environmental factors in the pathogenesis of melasma. Specifically, the observed associations between alcohol consumption, breast cystic hyperplasia, and melasma suggest that systemic health conditions may play a considerable role in the development of skin disorders. In contrast, the protective effects associated with relocation and house renovation emphasize the importance of environmental improvements in reducing the risk of melasma.

Moreover, our focus on a Chinese population—a demographic often underrepresented in prior research—offers valuable insights into the complex etiology of melasma. By using a robust sample size and conducting a comprehensive analysis of both traditional and emerging risk factors, our study adds depth to the understanding of this multifactorial condition. However, we acknowledge that the localized nature of our study and its relatively narrow scope may limit the generalizability of our findings. To confirm and expand upon these results, future research should involve larger, multi‐center studies across more diverse populations. Such studies would help determine whether our findings hold true across different demographic and geographic contexts.

We also recognize potential limitations in our matching criteria. While our study matched case–control participants based on gender, age, and body mass index (BMI), melasma is influenced by a range of other factors, including skin phototype, UV exposure, and hormonal status, which were not included in our matching process. Despite our efforts to account for these variables through thorough data collection and statistical analyses, residual confounding may still affect the results. Future studies could improve methodological rigor by incorporating more comprehensive matching criteria and examining additional factors that contribute to melasma risk. Such improvements would increase the robustness and generalizability of the findings, further informing preventive and therapeutic strategies that target lifestyle and environmental modifications.

Although our study provides valuable insights into the etiology of melasma, it lacks detailed subclass analyses, such as the severity, types (e.g., epidermal, dermal, or mixed), chronicity, and the influence of previous treatments. These factors can significantly affect the pathogenesis, clinical presentation, and treatment outcomes of melasma. Including subclass analyses would enable a deeper understanding of the condition's heterogeneity and its response to various interventions. Future studies should consider stratifying patients according to these characteristics to identify potential differences in risk factors and outcomes across subgroups. This would help tailor prevention and treatment strategies to the specific needs of different patient populations, ultimately improving clinical management.

One major limitation of our study is its retrospective case–control design, which may lead to spurious correlations and contradictory findings due to biases such as recall and selection bias. While we employed statistical methods to mitigate these issues, the inability of this design to establish causal relationships remains a significant limitation. To clarify these relationships and validate our findings, prospective comparative studies are necessary. Such studies would provide stronger evidence by allowing better control over confounding variables and offering clearer insights into the causal mechanisms linking identified risk and protective factors with melasma.

Our study's focus on a single‐center cohort in a specific locality may further restrict the generalizability of our findings to other regions or ethnic groups in China. Given the country's diverse genetic backgrounds, environmental exposures, and cultural practices, similar studies conducted in different regions and among various ethnic populations would offer more robust evidence. These studies could identify regional and ethnic variations in risk factors, which would facilitate the development of more targeted prevention and treatment strategies for melasma.

## 5 Conclusion

This case–control study uncovered for the first time that alcohol intake, breast cystic hyperplasia, change of residence, house renovation and soft drink intake are linked to morbidity of melasma in the Chinese population. These results indicate that diet and living environment are involved in its etiology. In clinical practice, it is critical for dermatologists to pay more attention to these novel factors and provide each patient with targeted guidance.

## Author Contributions

Cheng Tan contributed to the conception design and review. Shun Guo assisted with the conception design and was responsible for the data collection. Yue Shi performed the data analyses and wrote the manuscript.

## Ethics Statement

The study was conducted in accordance with the principles of the Declaration of Helsinki. The Jiangsu Province Hospital of Chinese Medicine Ethics Committee approved the study protocol (Ethical approval No. 2014NL‐024‐02), and all study subjects signed the informed consent form.

## Conflicts of Interest

The authors declare no conflicts of interest.

## Data Availability

The data that support the findings of this study are available from the corresponding author upon reasonable request.
